# Evidence of Distinct Tumour-Propagating Cell Populations with Different Properties in Primary Human Hepatocellular Carcinoma

**DOI:** 10.1371/journal.pone.0021369

**Published:** 2011-06-23

**Authors:** Federico Colombo, Francesca Baldan, Silvia Mazzucchelli, Ines Martin-Padura, Paola Marighetti, Alessandra Cattaneo, Barbara Foglieni, Marta Spreafico, Silvana Guerneri, Marco Baccarin, Francesco Bertolini, Giorgio Rossi, Vincenzo Mazzaferro, Massimiliano Cadamuro, Marco Maggioni, Luca Agnelli, Paolo Rebulla, Daniele Prati, Laura Porretti

**Affiliations:** 1 Experimental Hepatology Laboratory, Centre of Transfusion Medicine, Cellular Therapy and Cryobiology, Fondazione IRCCS Ca' Granda Ospedale Maggiore Policlinico, Milan, Italy; 2 Laboratory of Hemato-Oncology, European Institute of Oncology, Milan, Italy; 3 Department of Hematology, Hospital A. Manzoni, Lecco, Italy; 4 Laboratory of Medical Genetics, Fondazione IRCCS Ca' Granda Ospedale Maggiore Policlinico, Milan, Italy; 5 Liver and Lung Transplant Unit, Fondazione IRCCS Ca' Granda Ospedale Maggiore Policlinico, Milan, Italy; 6 Gastro-Intestinal Surgery and Liver Transplantation, National Cancer Institute, Milan, Italy; 7 Department of Surgical and Gastroenterological Sciences, University of Padua, Padua, Italy; 8 Center for Liver Research (CeLiveR), Ospedali Riuniti di Bergamo, Bergamo, Italy; 9 Division of Pathology, Department of Medicine, Surgery and Dentistry, A.O. San Paolo Milan, Milan, Italy; 10 Division of Pathology, Department of Medicine, Surgery and Dentistry, Fondazione IRCCS Ca' Granda Ospedale Maggiore Policlinico, Milan, Italy; 11 Department of Medical Sciences, University of Milan, Milan, Italy; MRC, University College of London, United Kingdom

## Abstract

**Background and Aims:**

Increasing evidence that a number of malignancies are characterised by tumour cell heterogeneity has recently been published, but there is still a lack of data concerning liver cancers. The aim of this study was to investigate and characterise tumour-propagating cell (TPC) compartments within human hepatocellular carcinoma (HCC).

**Methods:**

After long-term culture, we identified three morphologically different tumour cell populations in a single HCC specimen, and extensively characterised them by means of flow cytometry, fluorescence microscopy, karyotyping and microarray analyses, single cell cloning, and xenotransplantation in NOD/SCID/IL2Rγ^−/−^ mice.

**Results:**

The primary cell populations (hcc-1, -2 and -3) and two clones generated by means of limiting dilutions from hcc-1 (clone-1/7 and -1/8) differently expressed a number of tumour-associated stem cell markers, including EpCAM, CD49f, CD44, CD133, CD56, Thy-1, ALDH and CK19, and also showed different doubling times, drug resistance and tumorigenic potential. Moreover, we found that ALDH expression, in combination with CD44 or Thy-1 negativity or CD56 positivity identified subpopulations with a higher clonogenic potential within hcc-1, hcc-2 and hcc-3 primary cell populations, respectively. Karyotyping revealed the clonal evolution of the cell populations and clones within the primary tumour. Importantly, the primary tumour cell population with the greatest tumorigenic potential and drug resistance showed more chromosomal alterations than the others and contained clones with epithelial and mesenchymal features.

**Conclusions:**

Individual HCCs can harbor different self-renewing tumorigenic cell types expressing a variety of morphological and phenotypical markers, karyotypic evolution and different gene expression profiles. This suggests that the models of hepatic carcinogenesis should take into account TPC heterogeneity due to intratumour clonal evolution.

## Introduction

Like many other solid tumours, hepatocellular carcinomas (HCCs) are characterised by a high degree of heterogeneity that is traditionally explained on the basis of one of two models of carcinogenesis: the stochastic model and the hierarchy model. The stochastic model predicts that a malignancy consists of a homogeneous population of cells that generate their heterogeneity and tumour-initiating potential in response to particular combinations of endogenous factors (gene dose effects, and transcriptional and translational control mechanisms) and exogenous factors (cytokine concentrations, cell-to-cell interactions and, particularly, a niche environment). The hierarchy model predicts that the organisation of a malignancy is similar to that of normal tissue hierarchy, with tumour-initiating cells producing identical daughter stem cells with a capacity for self-renewal and committed progenitor daughter cells with a limited (but potentially still significant) capacity to divide.

Over the last ten years, increasing evidence has been published indicating that tumour maintenance and growth are sustained by a minority population of tumour-propagating cells (TPCs) or cancer stem cells (CSCs) [1]–[5]. This is also true of liver cancer [6]–[8], and may have important diagnostic and therapeutic implications [9]. It was initially argued that the CSC model is essentially synonymous with the hierarchy model of carcinogenesis [10], but it has recently been suggested that it is compatible with both models [11] because “stemness” exists as a functional phenotype in the stochastic model and could be shown by any member of the malignant population in the presence of the appropriate endogenous and exogenous factors.

Efforts have recently been made to accommodate a further biological phenomenon in the models of carcinogenesis, because there is now convincing evidence that cancer cells are subject to a process known as clonal evolution: i.e. the continuous development of new clones characterised by new genetic (and possibly epigenetic) changes. Cancer cells constantly need to adapt to environmental pressures and these adaptations may affect their proliferation, metastatic potential or drug resistance, a process that can be reconciled with both the CSC and stochastic models of heterogeneity [11]. However, the clonal evolution model has not yet been fully applied to studies of liver CSCs, because most experiments have used clonally derived cancer cell lines that have been cultured for decades and therefore consist of relatively homogenous cell populations. In this regard, it is currently agreed that studies of TPCs should be extended to cells directly isolated from primary cancers [12].

In our early experiments, we isolated and expanded TPCs from primary human HCC specimens by adapting methods that have been successfully used to isolate normal liver stem cells [13]. The first samples came from small HCCs obtained after surgical resection, but we could not prolong epithelial cell cultures beyond the third or fourth passage. Based on our hypothesis that this was due to the small size of the specimens in relation to the total tumoral mass and the rarity of TPCs, we subsequently concentrated on larger nodules (>5 cm in diameter) and found that morphologically and phenotypically different cell populations could be cultured from the same tumour specimen. More specifically, three distinct and stable primary tumour propagating cell lineages (hcc-1, hcc-2 and hcc-3) obtained from a 74-year-old male patient with advanced HCC were expanded and extensively characterised by means of flow cytometry, fluorescence microscopy, karyotyping and microarray analyses, single cell cloning, and xenotransplantation in NOD/SCID/IL2Rγ^−/−^ (NSG) mice. The results indicate that the clonal evolution of TPCs is a driver of intra-HCC heterogeneity.

## Materials and Methods

### Ethics Statement

The study patients gave their written informed consent before undergoing HCC resection, and the study was approved by the Institutional Review Board of the Fondazione IRCCS Ca' Granda Ospedale Maggiore Policlinico. Experiments involving animals were done in accordance with the Italian Laws (D.L.vo 116/92 and following additions, which enforces EU 86/609 Directive).

According to the regulatory requirements, the European Institute of Oncology animal facility is fully authorized by the Italian Ministry of Health (DM N° 65/2007-A) and the project has been notified to the Ministry of Health with ID number 11/09.

### Liver cell isolation and long-term cell cultures

After appropriate samples had been taken for histological purposes, a liver tumour tissue specimen was collected and dissociated as previously described [13].

The cell suspensions obtained from the tumoral tissue were cultured onto collagen-coated Petri dishes at a density of 5×10^5^ cells/cm^2^ in IMDM, supplemented with 20% FBS, 1% non-essential amino acids, 1% glutamine, and 1% penicillin/streptomycin (Invitrogen, Carlsbad, CA, USA). The medium was changed 24 hours after seeding in order to remove dead cells and debris, and was then replaced twice a week; the cells were maintained at 37°C in a humidified 5% CO_2_ incubator. After the appearance of colonies of 50–100 cells (usually after 14–20 days), the cells were replated in plastic flasks. Colonies with different cell morphologies were picked up separately and replated in different flasks. Confluent cells were detached using trypsin/EDTA (Invitrogen), counted and replated 1∶3 at every split in order to determine their growth kinetics.

Three morphologically different cell colonies (hcc-1, hcc-2 and hcc-3) were raised in culture from a single specimen of about 15 grams taken from a male patient with trabecular HCC who was HBsAg and anti-HCV negative and had no clinical or histological evidence of liver cirrhosis.

### In vitro clonogenicity

In order to determine whether heterogeneity was an intrinsic property of the three cell populations, each one was plated as a single cell by means of limiting dilution in 96-well plates. Clones with >50 cells were scored after three weeks, picked up and plated alone in flasks; cloning efficiency was defined as the percentage of cells developing a clone. The clones were characterised by flow cytometry at the first and fifth culture passages, after which only those whose morphological and/or phenotypical profile were different from the original mother population were further characterised. Moreover, once we had evaluated the tumorigenicity *in vivo* of the three HCC cell lineages (see paragraph below) and since previous reports support the evidence that *in vitro* clonogenicity is related to *in vivo* tumorigenicity [7], [14]–[15], we performed single cell sorting on 96 well plates using FACSAria II equipped with FACSDiva software (BD) in order to evaluate the presence of a more clonogenic, and therefore theoretically more tumorigenic, subpopulation within the cell lines. In particular, for all the three HCC cell lines we sorted EpCAM positive and negative cells; moreover, we also sorted tumour cell lines choosing another typical specific marker for each of the three cell lines, i.e. CD44 for hcc-1, Thy-1 for hcc-2 and CD56 for hcc-3. In each experiment we chose to sort only the brightest and the most negative cells for each marker; these populations accounted for about 20% of each compartment. Cloning efficiency was evaluated as described above.

### Flow cytometry characterisation

The cells were characterised at passages 1–3, 9–15, and >30 as previously described [13], using the following monoclonal antibodies (moabs): anti-epithelial cell adhesion molecule (EpCAM), anti-Thy-1, anti-ATP binding cassette-G2 (ABCG2), anti-CD44, anti-CD49f , anti-CD56, anti-CD13, anti-CD166, anti-CD146, anti-CXCR4, anti-CD105, anti-platelet derived growth factor receptor-alpha (PDGFr-α) (Becton Dickinson Biosciences, BD, Franklin Lakes, NJ, USA), anti-CD133-1 (Miltenyi Biotec, Bergisch Gladbach, Germany), conjugated with fluorescein isothiocyanate (FITC) or phycoerythrin (PE) or PE-cyanin 7 (PE-Cy7) or allophycocyanin (APC) or APC-cyanin 7 (APC-Cy7). The fluorescence threshold between negative and positive cells was set on the basis of the reactivity of appropriate non-specific fluorochrome-conjugated isotypical controls.

Aldehyde dehydrogenase (ALDH) positivity was assessed, both alone or in combination with membrane-markers staining, on cell lineages using the Aldefluor kit (Stemcell Technologies, Vancouver, BC, Canada) in accordance with the manufacturer's instructions. At least 10^6^ cells were analysed using a FACSCanto II equipped with FACSDiva software (BD).

### Cell fluorescence microscopy

The HCC cell populations and clones were stained at the 15^th^ and fifth split respectively as previously described [13], using anti-cytokeratin (CK)18 (Sigma-Aldrich), anti-CK19 (Novocastra, Newcastle, UK), anti-albumin, anti-S100A4 (Dako, Glostrup, Denmark) and anti-ZO-1 antibodies (BD), and finally FITC or Texas Red-conjugated secondary antibodies (BD) specific to the appropriate species. After immunological staining, the nuclei were stained with 4,6-diamidino-2-phenylindole (DAPI, Sigma-Aldrich), and the images were taken using a Leica Microsystems DM IRE 2 microscope and analysed by means of FW4000I software (Leica Microsystems, Wetzlar, Germany).

### Immunohistochemistry of liver tissue

Formalin-fixed serially cut sections of primary tumour tissue were stained with hematoxylin and eosin (H&E) and evaluated for the expression of EpCAM (Santa Cruz, Santa Cruz, CA, USA), S100A4, CD56 and CK19 (all from Dako). The tissue staining and image analysis procedures have been described elsewhere [16].

### Cytogenetic studies

The tumour cells collected by means of trypsinisation at passages 3 and >30 were cytogenetically analysed. The cells were cultured (4 to 6 independent cultures were plated for each cell lineage and clone) for about a week, and then treated for three hours with colcemid solution. Chromosome preparations were set up using conventional techniques [17]. The metaphases were selected and captured by an ECLIPSE 400 fluorescent microscope (Nikon, Shinjuku, Japan), and the images were analyzed using Cytovision System version 2.03 for Windows (Genetix Ltd, New Milton, UK). Fluorescent *in situ* hybridization (FISH) was performed using conventional protocols [17] and a panel of probes specific for each chromosome (Multiprobe System-OctoChrome, Cytocell, Cambridge, UK).

### Gene expression analyses

Microarray expression was profiled by the Centre of Molecular Biomedicine Core Facilities (Trieste, Italy). The expression of more than 48,000 mRNA transcripts (representing 38,500 genes involved in cell cycle regulation, apoptosis, matrix adhesion and cell motility) was assessed by the HumanWG-6 v3 Expression BeadChip (Illumina, San Diego, CA, USA) in accordance with the manufacturer's instructions [18] (see Text S1). The data discussed in this article have been deposited in NCBI's Gene expression Omnibus (GEO) and are accessible through the accession number GSE24482 (http://www.ncbi.nlm.nih.gov/geo/query/acc.cgi?acc=GSE24482).

The abundance of selected transcripts previously identified by means of microarray expression profiling was re-evaluated using quantitative PCR (qPCR) [19] (see Text S1 and Table S1).

### Drug resistance assay

The cell populations and their clones were further characterised by analysing their resistance to the chemotherapeutic agent Sunitinib (formerly SU11248, Pfizer, New York, NY, USA), a multitargeted tyrosine kinase inhibitor with anti-angiogenic and anti-tumoral activities that has recently been evaluated in clinical trials as a treatment for HCC [20].

The cells were treated with 3, 6, 10, 13, 15 and 25 µM of Sunitinib dissolved in DMSO, and an MTT (3-(4,5 dimethylthiazol-2-yl)-2,5-diphenyltetrazolium bromide, Sigma-Aldrich) assay was used to determine the proportion of live cells 24 and 48 hours after treatment. IC_50_ values were calculated by means of non-linear regression analysis using GraphPad Prism version 5.0 software (GraphPad Software, San Diego, CA, USA), and the differences in the IC_50_ values were statistically analysed using two-way ANOVA on the basis of the results of at least three independent experiments with four replicates of each cell type per experiment. P values<.05 were considered statistically significant [21].

### In vivo tumorigenic potential

In order to verify whether the three cell populations and two clones contained TPCs, we performed serial xenotransplantation assays using 6–8 week old male NSG mice (Jackson Laboratory, Bar Harbor, ME, USA) kept under specific pathogen-free conditions [22]. In detail, 1×10^6^ hcc-1, -2 and -3 cells and 1×10^5^ hcc-1, clone-1/7 and -1/8 cells were directly injected into the liver parenchyma of the anesthetised mice after an abdominal incision. Since we previously observed a good correlation between human alphafeto-protein (AFP) plasma levels and tumor size measured by imaging [23], tail blood samples were collected and plasma AFP was evaluated using ARCHITECT 2000 (Abbott, Abbott Park, IL, USA) at various time points in order to estimate the tumour growth. Upon sacrifice, cardiac blood was collected, and the tumours and metastases were resected for further analysis.

## Results

### Long-term cultures

**Figure 1:**
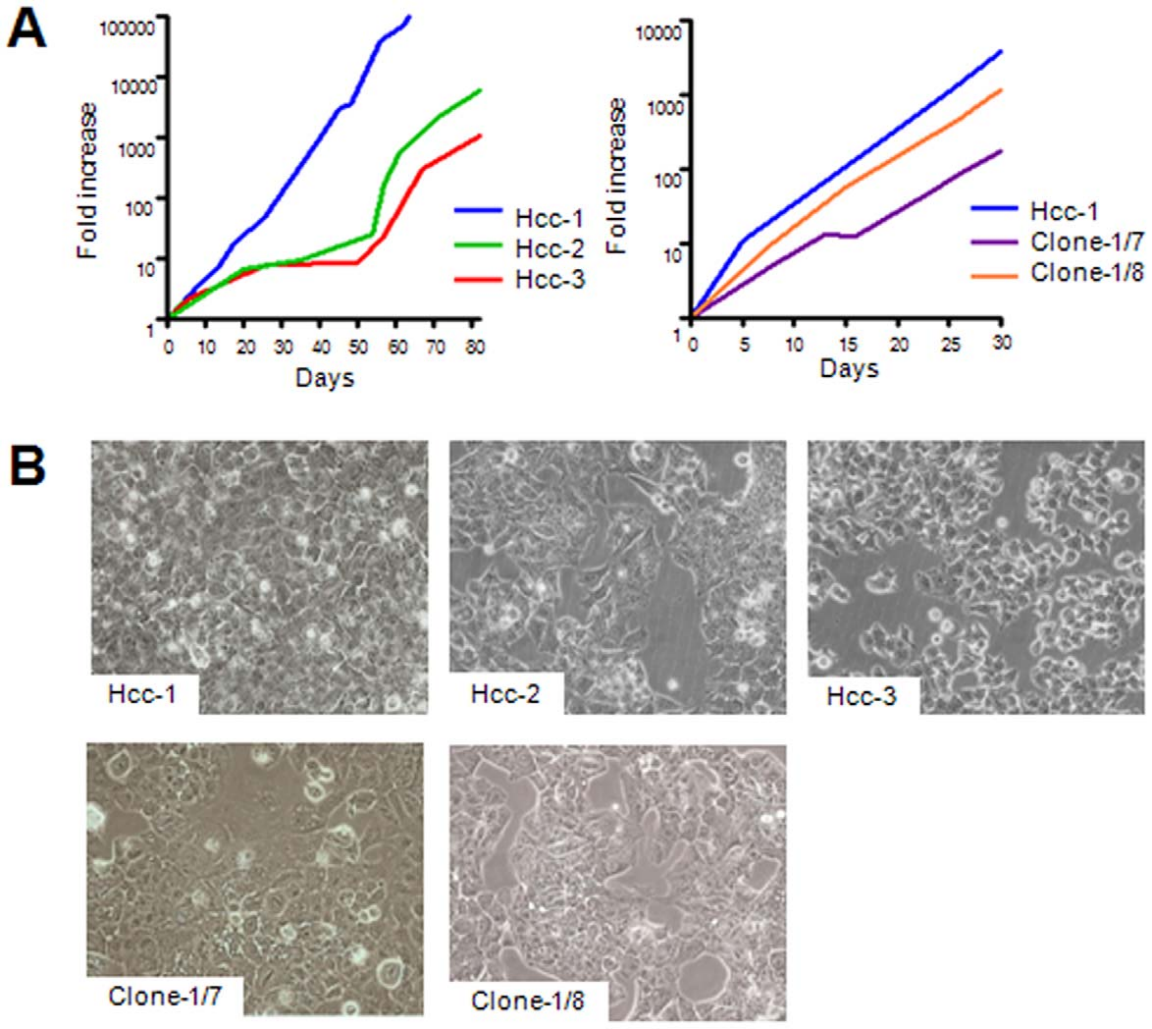
Different cell proliferation and morphology of the cell populations and clones generated from a single HCC specimen. A) Growth kinetic curves of the cell populations and clones. Hcc-1, hcc-2 and hcc-3 had doubling times of three, seven and six days respectively (left panel); hcc-1, clone-1/7 and clone-1/8 had doubling times of three, 4.7 and 3.6 days respectively (right panel). B) Representative phase-contrast images showing the morphology of the cell populations and clones; original magnification 20×.

Although they were maintained in long-term culture under the same conditions, the three primary cell lineages originating from a single HCC specimen had different growth kinetics and morphological patterns (Figs. 1A, 1B). In order to confirm that no cross-contamination with other samples had occurred, the three cell populations and frozen cells collected immediately after tumour dissociation underwent DNA Short Tandem Repeat (STR) analysis (Figure S1).

### In vitro clonogenicity

All of the cell populations were capable of generating clones by limiting dilution with a clonal efficiency of 14% for hcc-1, 12% for hcc-2, and 6% for hcc-3. Interestingly, all of the hcc-2 and hcc-3 clones were morphologically and phenotypically identical to their mother population, whereas hcc-1 generated at least two clones (clone-1/7 and clone-1/8) with different growth kinetics and morphologies from those of the mother cell population (Figs. 1A, 1B).

**Figure 2:**
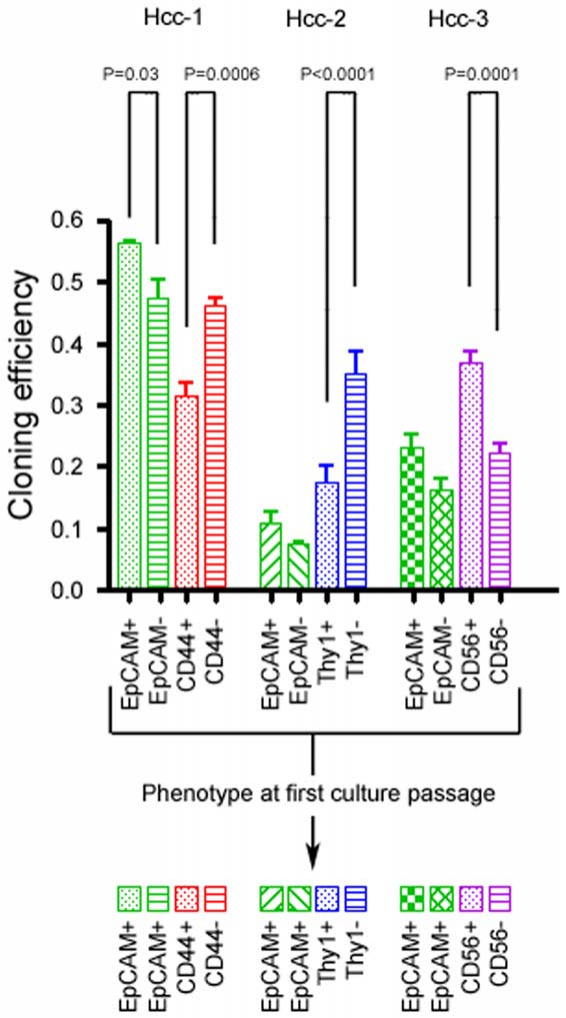
FACS cloning efficiency. Diagrams showing the different clonogenic potential of the 3 primary HCC cell lines sorted for specific membrane markers. Columns (mean and standard deviation of 3 independent experiments) represent the percentage of cells which gave rise to a colony. P<.05 was considered statistically significant (Chi square test). The lower part of the figure reports the expression of the sorting marker on each generated colony. All hcc-1, hcc-2 and hcc-3 colonies were positive for EpCAM regardless of whether they were generated by EpCAM positive or negative sorted cells. The colonies generated by sorting of the other specific markers, i.e. CD44, Thy-1 and CD56, did not show any modification of the expression of these markers.

Fluorescence activated single cell sorting evidenced a statistically higher clonogenic potential of EpCAM positive tumour cells only in hcc-1 cell line (p = .03, Chi square test). With regard to the other specific markers, hcc-1 CD44 negative cells, hcc-2 Thy-1 negative cells and hcc-3 CD56 positive cells were significantly more clonogenic than their counterparts (p = .0006, p<.0001 and p = .0001, respectively, Fig. 2). Once wells of the 96 well plates reached confluence, cells were evaluated for their marker expression. All hcc-1, hcc-2 and hcc-3 colonies were positive for EpCAM regardless of whether they were generated by EpCAM positive or negative sorted cells. On the contrary, colonies generated by CD44, Thy-1 and CD56 positive or negative cells did not shown any changes in the expression of these markers (see figure 2).

### Flow cytometry and fluorescence microscopy characterisation

**Figure 3:**
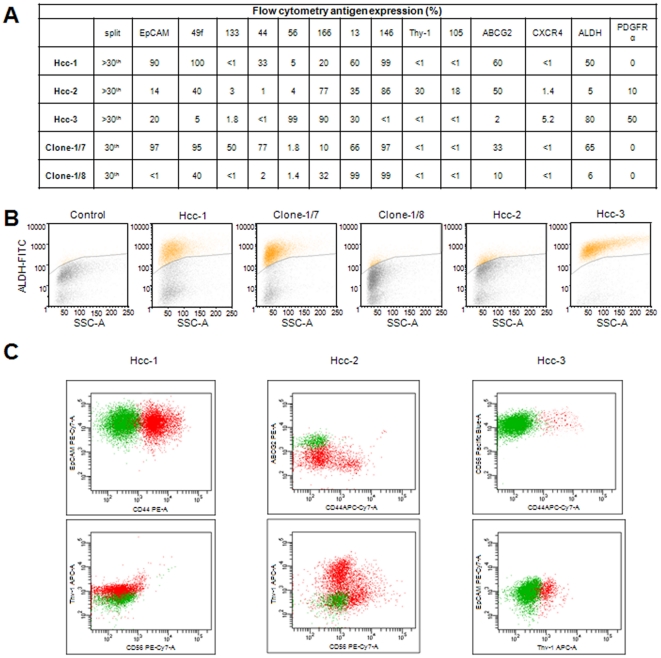
Flow cytometry characterisation of the HCC cell populations and clones in terms of the expression of the principal stem cell, epithelial and mesenchymal markers. A) Marker expression in the cell populations and clones, expressed as percentages of positive cells. B) ALDH expression in the cell populations and clones. C) Co-expression of the principal stem cell markers on ALDH positive (green dots) and negative (red dots) cells; hcc-1 left panels, hcc-2 middle panels and hcc-3 right panels, respectively.

Between the first and the tenth culture passages, the antigen expression pattern within each cell culture progressively stabilised. Figure 3 shows the different antigen expression in the cell populations and clones after the 30^th^ culture passage. The phenotypical profiles of hcc-1, -2 and -3 were significantly heterogeneous in terms of EpCAM, CD49f, Thy-1, CD56, CD44, CD146, ABCG2 and PDGFr-α expression (Fig. 3A); furthermore, the two clones differed from the mother hcc-1 cell line in terms of the expression of EpCAM, CD49f, CD133 and CD44 (Fig. 3A). ALDH activity also varied, being most highly expressed in hcc-3 followed by hcc-1; its expression was also higher in clone-1/7 than in clone-1/8 (Fig. 3B). ALDH was mainly expressed by CD44 negative cells in hcc-1, hcc-2 and hcc-3 cell lines; moreover, ALDH positivity was associated to Thy-1 negativity and ABCG2 positivity in hcc-2 and CD56 positivity in hcc-3 cells (Fig. 3C).

**Figure 4:**
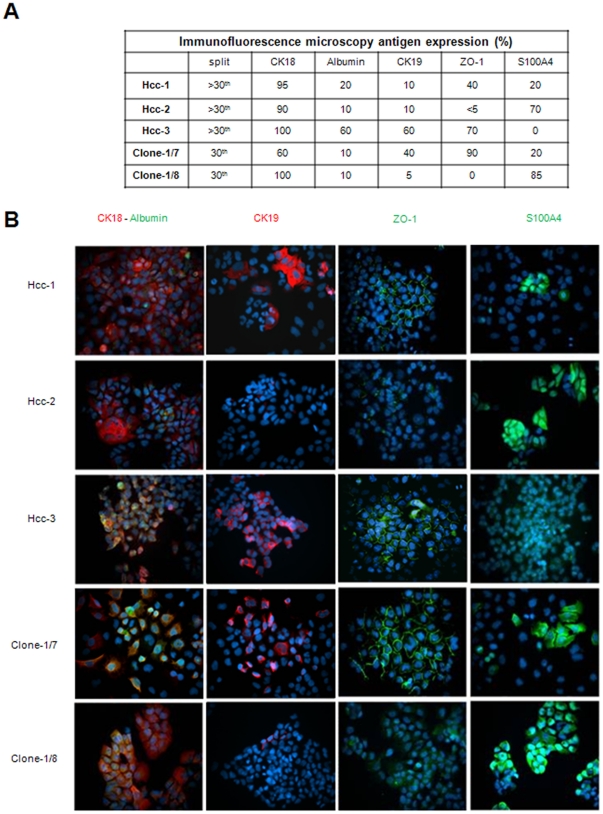
Immunofluorescence of cell culture. A) Double and single immunofluorescence staining of hcc-1, hcc-2, hcc-3, clone-1/7 and clone-1/8 using anti-CK18, -albumin, -CK19,-S100A4 and -ZO-1 antibodies. All nuclei were stained with DAPI (blue); original magnification 40×. B) Percentage of antigen expression in the three cell populations and clones.

Immunofluorescence microscopy showed that CK18 and albumin were expressed in all of the cell populations and clones, although the percentages of positive cells and the strength of the reactivity varied (Fig. 4A), whereas the expression of CK19 and ZO-1 was restricted to hcc-1, hcc-3 and clone-1/7. There was intense S100A4 staining in hcc-2 and clone-1/8, but hcc-3 did not express the protein (Fig. 4B).

### Tissue immunohistochemistry

**Figure 5:**
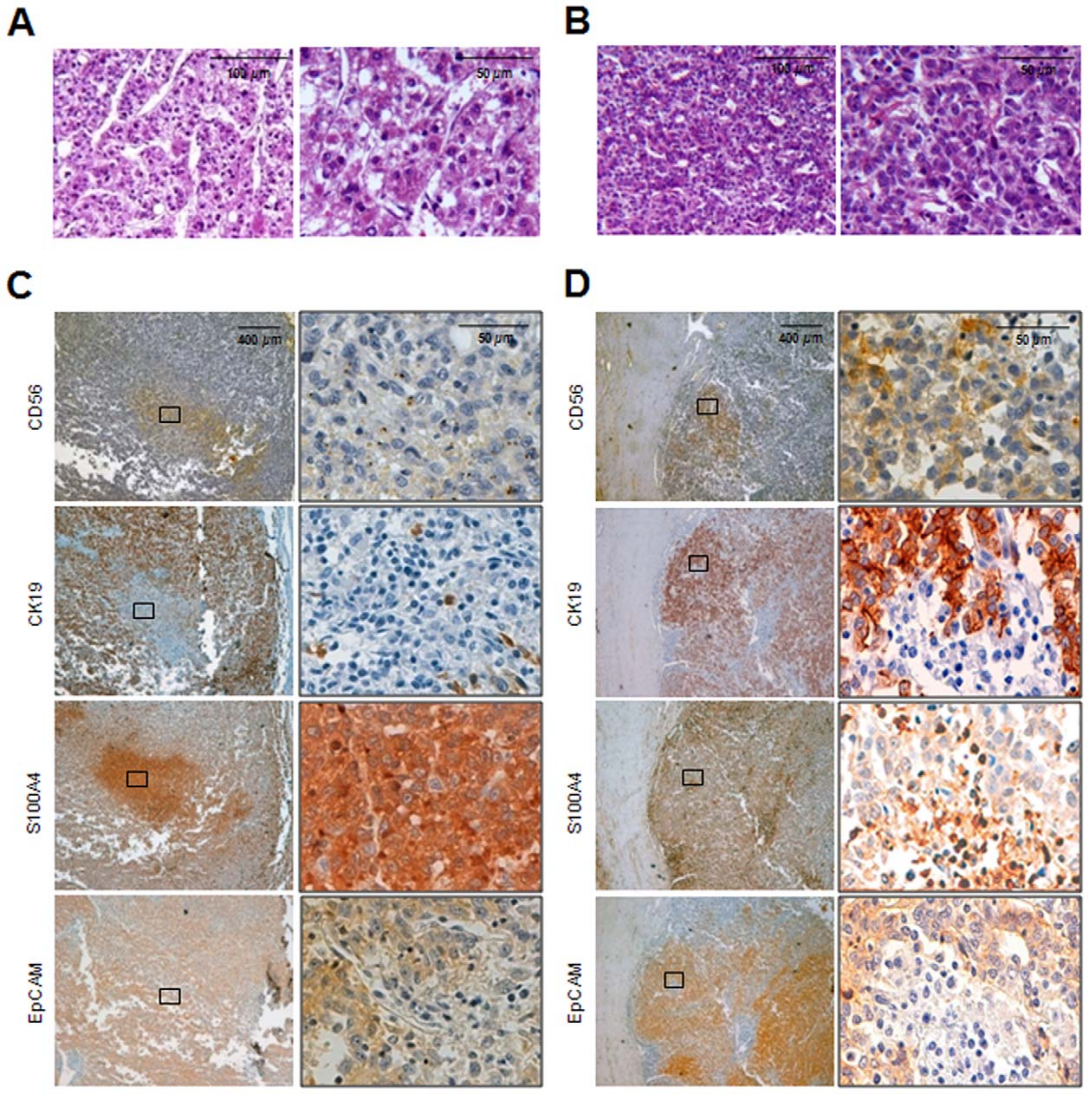
Immunohistochemistry of primary HCC tissue. The staining was chosen on the basis of the characteristic markers of the three cell populations detected in culture: CD56, CK19, S100A4 and EpCAM. A and B) H&E staining showing cell morphology heterogeneity in different tumour areas. C) Left panels: serial tissue sections showing areas with the phenotype resembling hcc-1 (CD56−/CK19+/S100A4−/EpCAM+) and hcc-2 (CD56−/CK19−/S100A4+/EpCAM+/−); right panels: higher magnifications of the areas indicated by the black rectangles. D) Left panels: serial tissue sections showing an area resembling the phenotype of hcc-3 (CD56+/CK19+/S100A4−/EpCAM+); right panels: higher magnifications of the areas indicated by the black rectangles.

Morphological cell evaluations of different tissue sections revealed the presence of some micro nodules with small round cells with little cytoplasm; the external tumoral tissue forming most of the tumour bulk had larger cells resembling typical mature epithelial cells (Figs. 5A, 5B). This heterogeneity was confirmed by the immunological staining of serial tumor sections. In particular, as in the case of the immunofluorescence staining, there was a micro nodule that was positive for S100A4 but negative for CD56 and CK19, and another that was positive for CD56 and CK19 but negative for S100A4 (Figs. 5C, 5D). Several examined sections showed widespread staining indicating EpCAM tissue expression, with the more or less intensely stained areas approximately corresponding to the S100A4 negative or positive nodules (see in particular fig. 5D and Figure S2).

### Genomic aberrations

**Figure 6:**
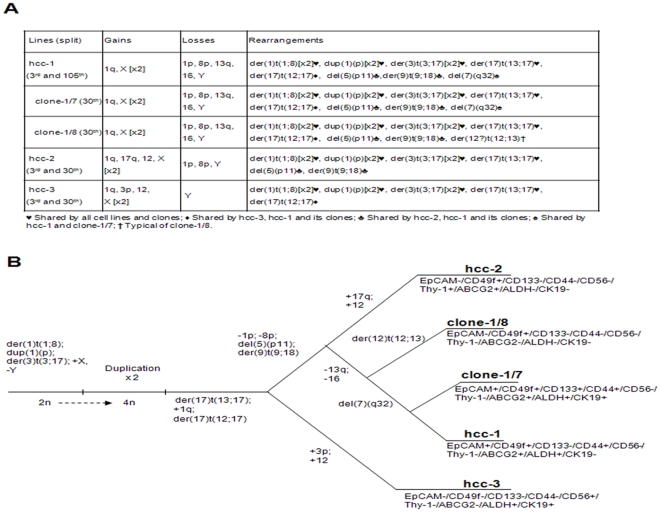
Clonal genomic alterations in the cell populations and clones. A) Gains, losses and rearrangements in the three populations observed at both the 3^rd^ and >30^th^ culture passages, thus indicating that the main clonal chromosomal abnormalities are likely to have been present in the primary tumour. Clone-1/7 and 1/8 were examined at the 30^th^ culture passage. B) Dendrogram reproducing the development of genomic alterations and the possible clonal evolution of the populations and clones. Hcc-2 probably lost the der(17)t(12;17) during its evolution process. For each cell lineage and clone the stem cell marker status is reported. The signs plus (+) and minus (−) mean that more or less than 30% of the cells were positive or negative for that marker, respectively.

Karyotyping and FISH analyses showed that the three cell populations had complex hypotetraploid karyotypes (Figure S3). As expected, the populations and clones shared many common clonal alterations as they were cultured from the same tumour sample, but they also had some distinctive features (Fig. 6A). All of them shared translocation t(1∶8) and the gain of 1q, which may be early genomic alterations in HCC development [24]: the first may confer a growth advantage [25] and the second contains several candidate oncogenes [24]. Loss of 1p and 8p was only observed in the hcc-2 and hcc-1 cells and the clones, thus suggesting a more advanced occurrence [26]; the loss of 13q was only observed in hcc-1 and its clones, which suggests a late onset [27].

The alterations accumulating from hcc-3 to hcc-1 could be fitted in a dendrogram showing the hypothetical clonal evolution of the cell populations and the clones (Fig. 6B). The presence of the same chromosomal abnormalities in more than two independent cultures of each cell lineage and clone, together with the presence of the same chromosomal alterations at both early (3^rd^ split) and late culture passages (>30^th^ split), indicated that they probably had not arisen *in vitro*.

### Microarray and qPCR

**Figure 7:**
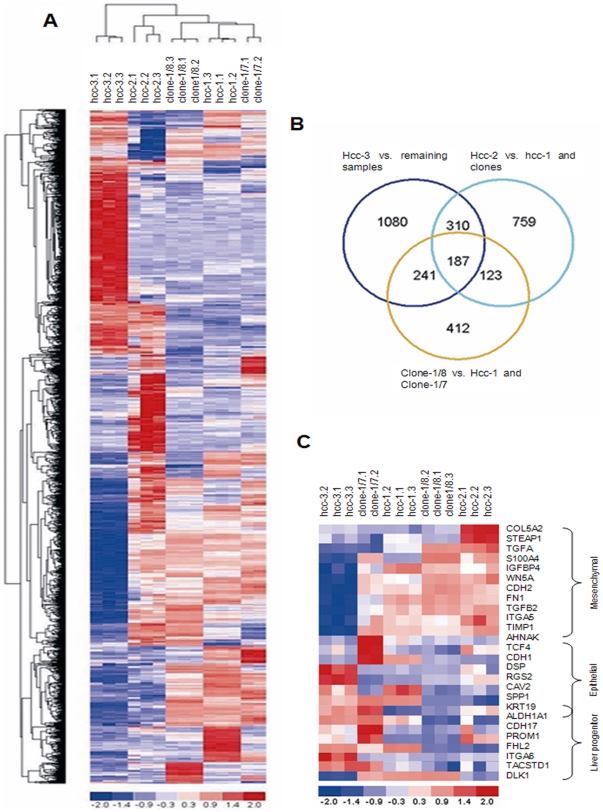
Microarray results of whole genome analysis. A) Heat map showing the clusterization of lineages and clones based on the expression profile of the 1196 most variable genes across the data set. B) Venn diagram indicating the distribution of the 3112 genes differently expressed in the supervised analyses. C) Heat map showing differently expressed levels of liver progenitor, epithelial and mesenchymal genes among HCC lineages and clones. Lineages and clones are arranged to evidence the transition from an epithelial expression pattern to a mesenchymal one.

Unsupervised analyses of the 1196 most variable genes across the dataset generated a dendrogram showing, as expected, a low dissimilarity among hcc-1 and its clones, while hcc-2 showed higher differences and hcc-3 had the most different expression profile (Fig. 7A).

Supervised analyses of hcc lineages and clones performed on the 16964 normalized expression values permitted to generate a Venn diagram showing the distribution of the 3112 genes that resulted differently expressed among the different comparisons (Fig. 7B). We evidenced that the 187 genes that resulted differentially espressed in all comparisons are enriched in genes involved in cell lipid catabolism, peptidase inhibitor activity, glycoprotein biosynthesis, regulation of transcription, and extracellular and membrane components.

The heatmap of “epithelial-to-mesenchymal transition” [28] (EMT) genes revealed that hcc-3, followed by hcc-1, expressed high levels of progenitor and epithelial genes, whereas hcc-2 expressed higher levels of the mesenchymal ones. Similarly, clone-1/7 expressed higher levels of epithelial progenitor genes and lower levels of the EMT ones, while clone-1/8 had a more mesenchymal pattern of gene expression than hcc-1 *in toto* (Fig. 7C).

Quantitative PCR of seven genes characteristic of the cell populations and clones showed the same expression pattern as that revealed by the microarrays (Figure S4).

### Resistance to sunitinib

**Figure 8:**
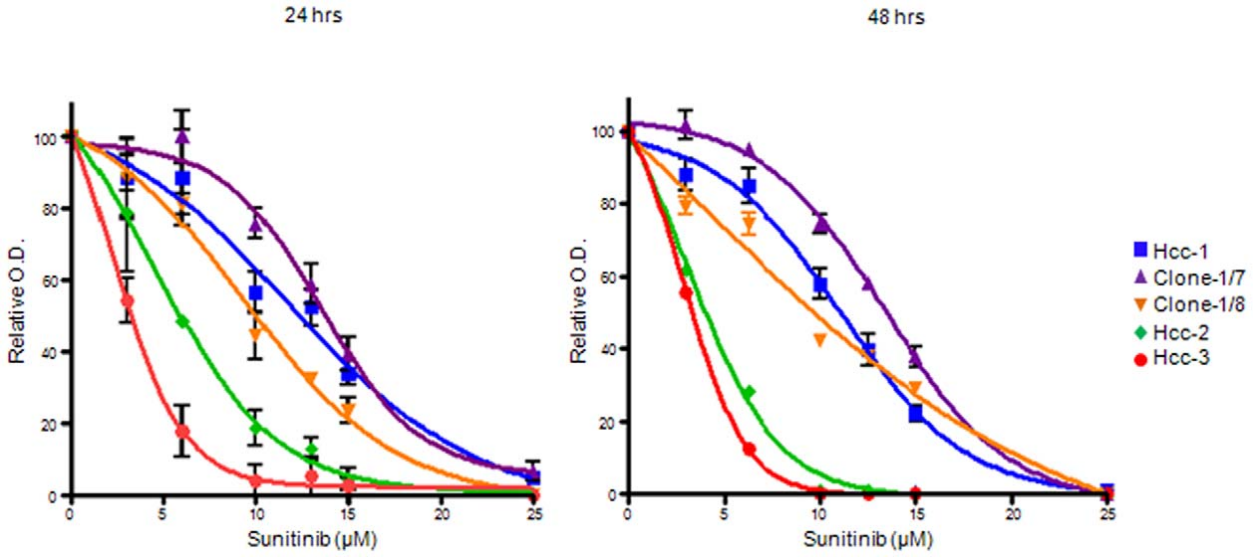
Drug resistance of the cell populations and clones. The MTT evaluation showed that the cell populations and clones had different chemoresistance to sunitinib after 24 (left) and 48 (right) hours of treatment. Mean ± standard deviation of three independent experiments.

Hcc-1 showed greater resistance to sunitinib than hcc-2 or hcc-3 after both 24 and 48 hours of treatment at concentrations of >3 µM (P<.01, 2-way ANOVA), whereas there were no differences between hcc-2 and hcc-3 at any concentration or time point. Interestingly, clone-1/7 was more resistant than clone-1/8 or hcc-1 *in toto* at each concentration after both 24 and 48 hours (P<.001); on the contrary, clone-1/8 was less resistant than hcc-1 *in toto* after 24 hour treatment with 6 µM (P<.05) but remained different after 48 hours of treatment only at sunitinib doses of 3–10 µM (P<.05, Fig. 8).

### Tumorigenic potential

In order to test the capacity of the cell populations to initiate *in vivo* tumour formation, we carried out intra-hepatic transplantations into NSG mice. As the primary HCC secreted AFP, we monitored blood AFP levels during the post-transplant period and, when the mice showed high AFP levels and/or signs of illness, they were immediately sacrificed and examined for tumour formation.

**Figure 9:**
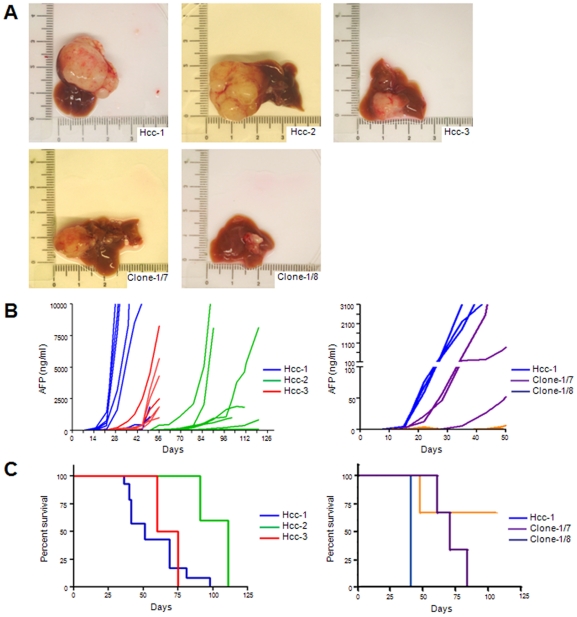
Tumorigenicity in vivo. A) Representative liver tumours generated in NSG mice injected with the three cell populations and clones. B) Human AFP detection in blood from mice inoculated with 1×10^6^ hcc-1, -2 and -3 cells (left; n = 21, 7 per group) and with 1×10^5^ hcc-1, clone-1/7 and clone-1/8 (right; n = 9, 3 per group). C) Different rate of mouse survival after hcc-1, -2, -3 inoculations (left; P = .012) and hcc-1, clone-1/7 and clone-1/8 inoculations (right; P = .08).

All of the mice injected with hcc-1, -2 and -3 (n = 21; seven per cell population) developed hepatic tumours (Fig. 9A) and/or abdominal tumours, but the time courses were different. The mice inoculated with hcc-1 had detectable AFP levels seven days after the injection, whereas those injected with hcc-2 or hcc-3 did not have detectable AFP levels until 56 and 22 days after the injection respectively (Fig. 9B).

The mice transplanted with hcc-1 had a shorter median survival (51 days) than those receiving hcc-2 or hcc-3 (111 and 67.5 days respectively; P = .012 by Kaplan-Meier survival analysis) (Fig. 9C). Interestingly, hcc-1 was more tumorigenic than its clones, as shown by the size of the generated tumour and the different AFP levels measured 20 days after the injection (Figs. 9A, 9B). Moreover, clone-1/8 generated a detectable liver tumour in only one of the three transplanted mice, and its AFP levels measured 35 days after the injection were significantly lower than those of the other clone or hcc-1 (0±0, 185±183 and 2390±694 ng/ml respectively; P = .0007, one way ANOVA) (Fig. 9B).

The dissociated xenografted tumours arising from the three cell populations generated cell cultures that fully recapitulated the phenotypical profile of the injected cells, and also generated secondary tumours if retransplanted into NSG mice (data not shown).

## Discussion

We isolated and expanded *ex vivo* three different cell populations from a single mass of advanced HCC after enzymatic dissociation of a highly representative portion of the total tumour mass (>80%). Single cell cloning experiments showed that hcc-1 was not a homogeneous lineage, but contained at least two distinct subpopulations (clone-1/7 and -1/8). However, all of the lineages were capable of forming clones that gave rise to progenies with the same immunophenotypical characteristics as those found in the original cultures.

Under identical culture conditions, the three populations and two clones had distinct morphological, phenotypical and gene expression profiles. Their growth patterns, the clonogenic assays and the xenotransplantation experiments clearly indicated that they all had tumour propagating capability, although the efficacy varied. Moreover, the xenografted tumour cells were capable of regenerating the originally inoculated populations, and maintained their tumorigenic potential and distinct features in secondary transplantations.

Karyotyping and FISH analyses showed that hcc-1, -2 and -3 and clone-1/8 were genetically distinct and, on the basis of their patterns of clonal abnormalities, we tracked their hypothetical evolution process. The oldest was hcc-3, followed by hcc-1 and hcc-2. Furthermore, our data agree with the current models of the step-wise progression of hepatocarcinogenesis [26]–[27], according to which the gain of 1q (present in all three cell populations) is a marker of early HCC, whereas losses of 8p (as in hcc2 and hcc1) and 13q (as in hcc-1 and its clones) are associated with clinical progression.

Nodules whose phenotypical characteristics were concordant with the three cell populations were clearly identifiable in the primary tumour tissue. Hcc-3 may have originated from a CD56^+^/CK19^+^/EpCAM^+^ microscopic nodule, and hcc-2 from a S100A4^+^/CK19^−^/CD56^−^ microscopic nodule; the majority of the tumour mass (which was negative for CD56 and S100A4 but expressed EpCAM and CK19) generated hcc-1, the most aggressive of the three cell populations. Other independent elements strongly indicate that the cell populations were not generated in culture: the three cell lineages remained functionally and phenotypically stable for more than one year after isolation; the genetic diversity of the populations was documented in the very early culture passages, with no new clonal chromosome abnormality occurring between the third and the last passage analyzed. Although we cannot completely exclude the occurrence of genetic changes between passage 1 and 3, this possibility seems unlikely based on previous reports [29]–[31]. Finally, as already mentioned, the hcc-1 population, which was the most representative population in the native tumour mass, was also the most phylogenetically rearranged.

Our data suggest that TPCs in the liver can be organised with a branching clonal architecture, and that a simple CSC model is not applicable to hepatocarcinogenesis. Our findings are novel in the field of liver carcinogenesis, but fit very well with previous observations in both hematological and solid tumours. Park *et al*. [32] have very recently documented the genetic heterogeneity of stem cell-like breast cancer cells within the same tumour. Preliminary experiments by Greaves' group [33] have demonstrated that the serial transplantation of genetically distinct clones of acute lymphoblastic leukemia cells from the same patient leads to the regeneration of leukemia in NSG mice at a level of genetic complexity that reflects the original diagnostic sample, which indicates that TPCs are genetically diverse. Finally, in an experimental setting very similar to our own, Piccirillo *et al*. [34] isolated two different populations of cancer cells from a single specimen of glioblastoma, which grew independently *ex vivo* and had different tumorigenic potential and genetic anomalies.

Morphological heterogeneity has long been recognised in HCC, and it has been recently shown that clinical HCC progression is related to the sequential accumulation of genomic aberrations [26]. In addition, clinical studies have shown that late HCC recurrences after surgery are frequently caused by clones that are distinct from those of the primary tumour [35]. It is possible that the heterogeneity of our patient's tumour mass was even greater than that described here because, as recently pointed out [34], other cell populations that are not capable of growing in culture may have been eliminated upon cell isolation.

However, our data are not irreconcilable with a more complex CSC hypothesis. As recently underlined [36], a comprehensive model combining the stem cell and clonal evolution theories simply moves the dynamics of intra-tumour evolution from the cancer cell population as a whole to the stem cell compartment. Our different populations contained elements with stem cell properties [9], including self renewal, differentiation, and tumour initiation capacity. They also contained (with different patterns and frequencies) the markers that have been previously identified in CSCs of epithelial tumours, including EpCAM, CD49f, CD133, CK19, CD44, ABCG2 and ALDH.

The evidence that only a minority of cells within the 3 cell lineages had cloning capacity (between 6 and 14%) suggested that, as already reported, tumour propagating ability may be restricted only to certain elements. Single cell sorting experiments showed different clonogenicity according to the expression of CD44, Thy-1 and CD56 antigens with a clear relation between the expression of these markers and ALDH activity which deserves further studies.

The different cell populations had different chemoresistance profiles. Resistance to sunitinib was directly related to tumorigenicity, and inversely related to the degree of membrane expression of PDGFr-α, one of the drug's main targets. Clonal organisation of a tumour may greatly affect treatment outcome because neoplastic evolution tends to select TPCs with treatment resistant features [37]. We also observed that hcc-3 and clone-1/7 were oriented towards a liver progenitor phenotype (EpCAM, ZO-1, CK19, ALDH, CD56 positivity); hcc-2 and clone-1/8 had the typical features of EMT (Thy-1, CD105, S100A4 positivity); and hcc-1, which had a mature epithelial morphology, had an intermediate phenotype that included both epithelial and mesenchymal markers. These data were also confirmed by differential gene expression analysis.

Although it is known that EMT in liver cancer is facilitated by genomic cell alterations [38], we cannot exclude the possibility that the EMT characteristics were also partially acquired *ex vivo* as an adaptation to culture conditions. However, the phenotypical features of the cell populations remained stable even after xenotransplantation, which makes this less likely.

We can only speculate on the different mechanisms that may maintain and promote this intra-tumour clonal heterogeneity because experimental evidence in humans is very limited [32], [37]. It is currently agreed that spatial restrictions in solid tumours lead to the generation of separate niches that favour the growth of cells with different characteristics. In addition, various types of cooperation among tumour cell populations have been proposed, including parasitism and commensalism [37]. Interestingly, the hcc-1 cells (which had both epithelial and mesenchymal features) were characterized by greater tumorigenic potential and a higher growth rate than clone-1/7 (epithelial) or clone-1/8 (mesenchymal) alone. An educated hypothesis that deserves exploration in future studies is that EMT, which plays a crucial role in tumorigenesis and cancer progression [38]–[42], leads to cells that are not necessarily more tumorigenic *per se*, but can produce a suitable microenvironment for epithelial stem cell growth, and eventually co-migrate to a new organ and enhance the formation of metastases.

### Conclusion

In conclusion, we provide the first evidence that the same HCC mass can contain genetically distinct cell populations with independent tumour propagating capability, but significantly different phenotypical and functional characteristics. Our findings offer new perspectives for the design of a comprehensive model of hepatocarcinogenesis that takes into account the clonal evolution of TPCs, and the definition of novel therapeutic strategies for advanced HCC.

## Supporting Information

Figure S1 **STR analysis.** The primary dissociated tumour sample and hcc-1, hcc-2 and hcc-3 showed the same STR profile confirming that the cell populations originated from the same patient.
(TIF)

Figure S2 **Representative tissue sections showing the EpCAM and S100A4 staining pattern.** Broken lines evidences areas positive for EpCAM and less intensely positive for S100A4; continuous lines evidences areas positive for S100A4 and negative for EpCAM. Original magnification 5×.
(TIF)

Figure S3 **Representative karyotypes of the three cell populations and clones.** Some of the clonal alterations are indicated by arrows; the red arrows indicate the clonal alterations specific to clone-1/8.
(TIF)

Figure S4 **Gene expression validation.** A) Microarray and B) qPCR expression values of selected genes in the cell lines and clones. The bars represent the mean relative expression values of three independent RNA isolations analysed in triplicate. For the qPCR analyses, all of the genes were normalised to CYC as the reference housekeeping gene. All of the data are presented as log_2_ transformations of gene-normalised signals. The qPCR results showed the same consistent modulation as the microarray analysis.
(TIF)

Table S1 **Primer and probe sequences used in the qPCR experiments.**
(TIF)

Text S1 **Supplementary Materials and Methods.**
(DOC)

## References

[1] Lapidot T, Sirard C, Vormoor J, Murdoch B, Hoang T (1994). A cell initiating human acute myeloid leukaemia after transplantation into SCID mice.. Nature.

[2] Al-Hajj M, Wicha MS, Benito-Hernandez A, Morrison SJ, Clarke MF (2003). Prospective identification of tumorigenic breast cancer cells.. Proc Natl Acad Sci USA.

[3] Singh SK, Hawkins C, Clarke ID, Squire JA, Bayani J (2004). Identification of human brain tumour initiating cells.. Nature.

[4] Dalerba P, Dylla SJ, Park IK, Liu R, Wang X (2007). Phenotypic characterization of human colorectal cancer stem cells.. Proc Natl Acad Sci USA.

[5] Visvader JE (2011). Cells of origin in cancer.. Nature.

[6] Chiba T, Kita K, Zheng YW, Yokosuka O, Saisho H (2006). Side population purified from hepatocellular carcinoma cells harbours cancer stem cell-like properties.. Hepatology.

[7] Ma S, Chan KW, Hu L, Lee TK, Wo JY (2007). Identification and characterization of tumorigenic liver cancer stem/progenitor cells.. Gastroenterol.

[8] Suetsugu A, Nagaki M, Aoki H, Motohashi T, Kunisada T (2006). Characterization of CD133+ hepatocellular carcinoma cells as cancer stem/progenitor cells.. Biochem Biophys Res Commun.

[9] Marquardt JU, Factor VM, Thorgeirsson SS (2010). Epigenetic regulation of cancer stem cells in liver cancer: current concepts and clinical implications.. J Hepatol.

[10] Shackleton M, Quintana E, Fearon ER, Morrison SJ (2009). Heterogeneity in cancer: cancer stem cells versus clonal evolution.. Cell.

[11] Bomken S, Fiser K, Heidenreich O, Vormoor J (2010). Understanding the cancer stem cell.. Br J Cancer.

[12] Chen R, Nishimura MC, Bumbaca SM, Kharbanda S, Forrest WF (2010). A hierarchy of self-renewing tumor-initiating cell types in glioblastoma.. Cancer Cell.

[13] Porretti L, Cattaneo A, Colombo F, Lopa R, Rossi G (2010). Simultaneous characterization of progenitor cell compartments in adult human liver.. Cytometry A.

[14] Kim Y, Lin Q, Zelterman D, Yun Z (2009). Hypoxia-regulated delta-like 1 homologue enhances cancer cell stemness and tumorigenicity.. Cancer Res.

[15] Shi GM, Xu Y, Fan J, Zhou J, Yang XR (2008). Identification of side population cells in human hepatocellular carcinoma cell lines with stepwise metastatic potentials.. J Cancer Res Clin Oncol.

[16] Fabris L, Cadamuro M, Libbrecht L, Raynaud P, Spirlì C (2008). Epithelial expression of angiogenic growth factors modulate arterial vasculogenesis in human liver development.. Hepatology.

[17] Chifenti B, Morelli M, Zavaglia M, Coviello DA, Guerneri S (2009). Establishment and characterization of 4 new human pancreatic cancer cell lines: evidences of different tumor phenotypes.. Pancreas.

[18] Agnelli L, Bicciato S, Fabris S, Baldini L, Morabito F (2007). Integrative genomic analysis reveals distinct transcriptional and genetic features associated with chromosome 13 deletion in multiple myeloma.. Haematologica.

[19] Livak KJ, Schmittgen TD (2001). Analysis of relative gene expression data using real-time quantitative PCR and the 2(-Delta DeltaC(T)) Method.. Methods.

[20] Faivre S, Demetri G, Sargent W, Raymond E (2007). Molecular basis for sunitinib efficacy and future clinical development.. Nat Rev Drug Discov.

[21] Liu L, Cao Y, Chen C, Zhang X, McNabola A (2006). Sorafenib blocks the RAF/MEK/ERK pathway, inhibits tumor angiogenesis, and induces tumor cell apoptosis in hepatocellular carcinoma model PLC/PRF/5.. Cancer Res.

[22] Agliano A, Martin-Padura I, Mancuso P, Marighetti P, Rabascio C (2008). Human acute leukemia cells injected in NOD/LtSz-scid/IL-2Rgamma null mice generate a faster and more efficient disease compared to other NOD/scid-related strains.. Int J Cancer.

[23] Martin-Padura I, Agliano A, Marighetti P, Porretti L, Bertolini F (2010). Sex-related efficiency in NSG mouse engraftment.. Blood.

[24] Lau SH, Guan XY (2005). Cytogenetic and molecular genetic alterations in hepatocellular carcinoma.. Acta Pharmacol Sin.

[25] Wong N, Lai P, Pang E, Leung TW, Lau JW (2000). A comprehensive karyotypic study on human hepatocellular carcinoma by spectral karyotyping.. Hepatology.

[26] Midorikawa Y, Yamamoto S, Tsuji S, Kamimura N, Ishikawa S (2009). Allelic imbalances and homozygous deletion on 8p23.2 for stepwise progression of hepatocarcinogenesis.. Hepatology.

[27] Steinemann D, Skawran B, Becker T, Tauscher M, Weigmann A (2006). Assessment of differentiation and progression of hepatic tumors using array-based comparative genomic hybridization.. Clin Gastroenterol Hepatol.

[28] Singh A, Settleman J (2010). EMT, cancer stem cells and drug resistance: an emerging axis of evil in the war on cancer.. Oncogene.

[29] Bigner SH, Mark J, Bigner DD (1987). Chromosomal progression of malignant human gliomas from biopsy to establishment as permanent lines in vitro.. Cancer Genet Cytogenet.

[30] Närvä E, Autio R, Rahkonen N, Kong L, Harrison N (2010). High-resolution DNA analysis of human embryonic stem cell lines reveals culture-induced copy number changes and loss of heterozygosity.. Nat Biotechnol.

[31] Rebuzzini P, Neri T, Mazzini G, Zuccotti M, Redi CA (2008). Karyotype analysis of the euploid cell population of a mouse embryonic stem cell line revealed a high incidence of chromosome abnormalities that varied during culture.. Cytogenet Genome Res.

[32] Park SY, Gönen M, Kim HJ, Michor F, Polyak K (2010). Cellular and genetic diversity in the progression of in situ human breast carcinomas to an invasive phenotype.. J Clin Invest.

[33] Greaves M (2010). Cancer stem cells: back to Darwin?. Semin Cancer Biol.

[34] Piccirillo SG, Combi R, Cajola L, Patrizi A, Redaelli S (2009). Distinct pools of cancer stem-like cells coexist within human glioblastomas and display different tumorigenicity and independent genomic evolution.. Oncogene.

[35] Hoshida Y, Villanueva A, Kobayashi M, Peix J, Chiang DY (2008). Gene expression in fixed tissues and outcome in hepatocellular carcinoma.. N Engl J Med.

[36] Merlo LMF, Maley CC (2010). The role of genetic diversity in cancer.. J Clin Invest.

[37] Merlo LMF, Pepper JW, Reid BJ, Maley CC (2006). Cancer as an evolutionary and ecological process.. Nat Rev Cancer.

[38] Kalluri R, Weinberg RA (2009). The basics of epithelial-mesenchymal transition.. Clin Invest.

[39] Villanueva A, Minguez B, Forner A, Reig M, Llovet JM (2010). Hepatocellular carcinoma: novel molecular approaches for diagnosis, prognosis, and therapy.. Annu Rev Med.

[40] Thiery JP, Acloque H, Huang RY, Nieto MA (2009). Epithelial-mesenchymal transitions in development and disease.. Cell.

[41] van Zijl F, Mair M, Csiszar A, Schneller D, Zulehner G (2009). Hepatic tumor-stroma crosstalk guides epithelial to mesenchymal transition at the tumor edge.. Oncogene.

[42] Vergara D, Merlot B, Lucot JP, Collinet P, Vinatier D (2010). Epithelial-mesenchymal transition in ovarian cancer.. Cancer Lett.

