# Supplementary figures and images for: Evidence of Distinct Tumour-Propagating Cell Populations with Different Properties in Primary Human Hepatocellular Carcinoma
Source: PLoS One. 2011 Jun 23;6(6):e21369. doi: 10.1371/journal.pone.0021369 (PMC3121782; doi:10.1371/journal.pone.0021369)

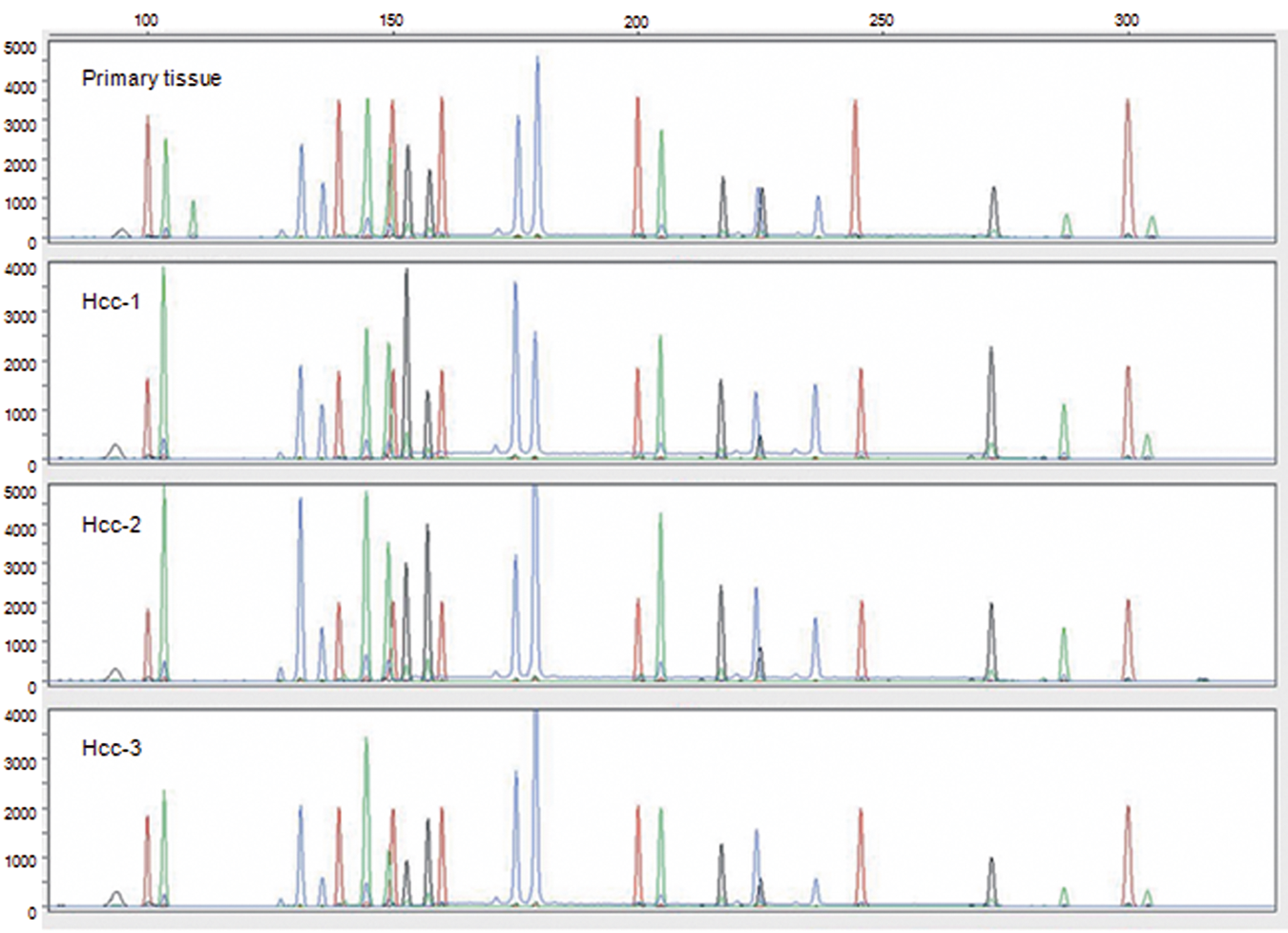

Supplement: Figure S1 — STR analysis. The primary dissociated tumour sample and hcc-1, hcc-2 and hcc-3 showed the same STR profile confirming that the cell populations originated from the same patient. (TIF) [file pone.0021369.s001.tif]

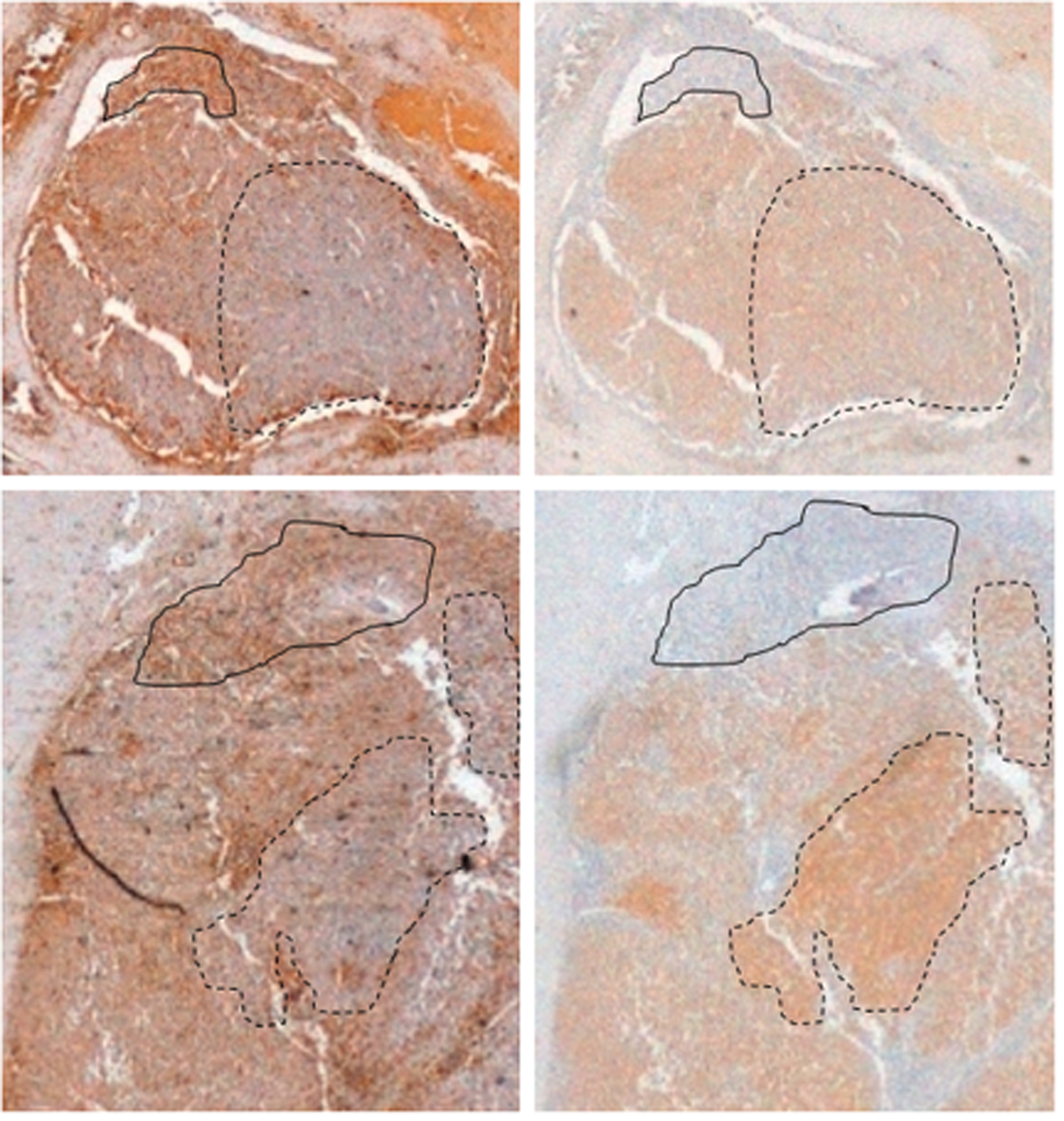

Supplement: Figure S2 — Representative tissue sections showing the EpCAM and S100A4 staining pattern. Broken lines evidences areas positive for EpCAM and less intensely positive for S100A4; continuous lines evidences areas positive for S100A4 and negative for EpCAM. Original magnification 5×. (TIF) [file pone.0021369.s002.tif]

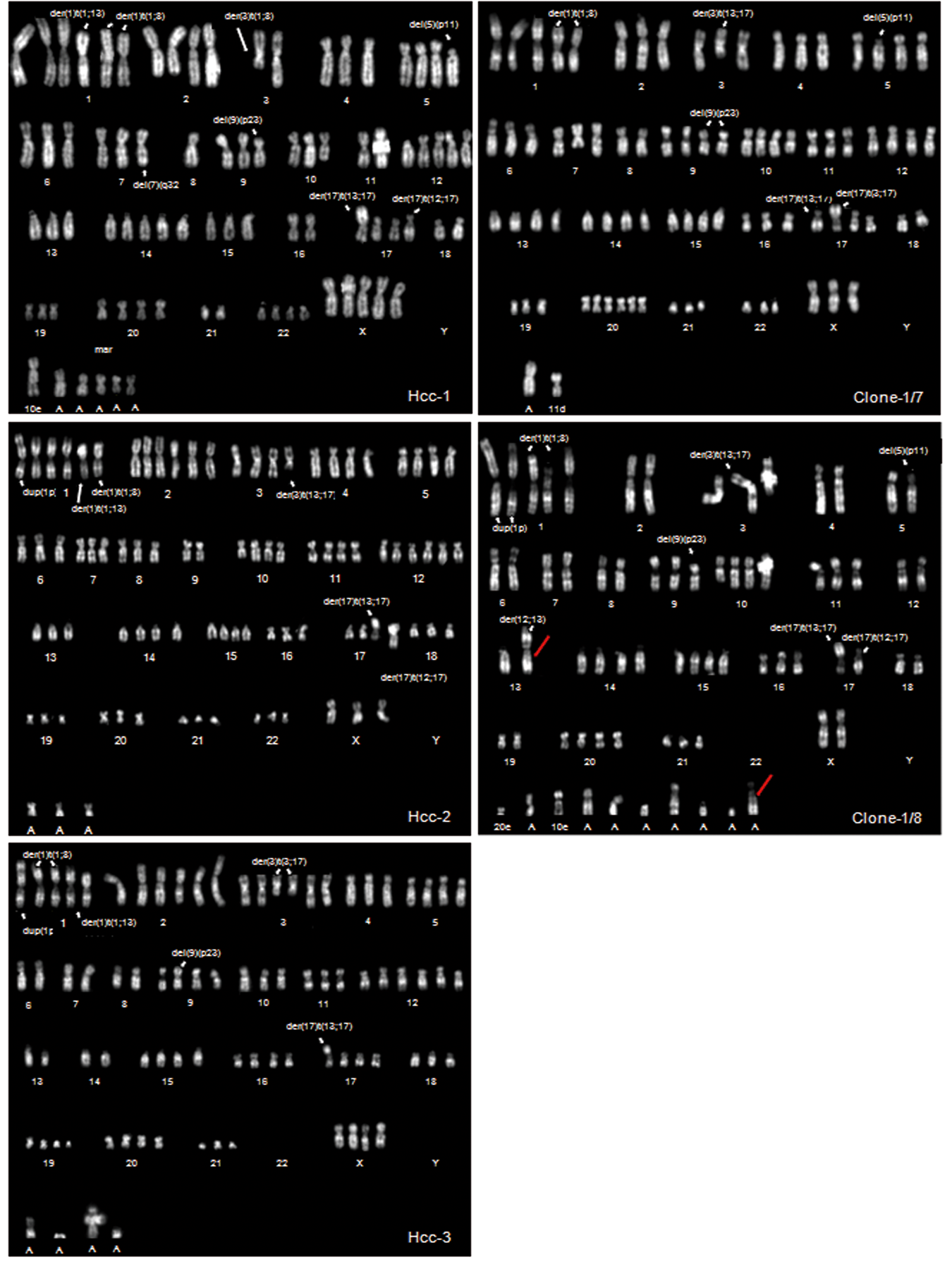

Supplement: Figure S3 — Representative karyotypes of the three cell populations and clones. Some of the clonal alterations are indicated by arrows; the red arrows indicate the clonal alterations specific to clone-1/8. (TIF) [file pone.0021369.s003.tif]

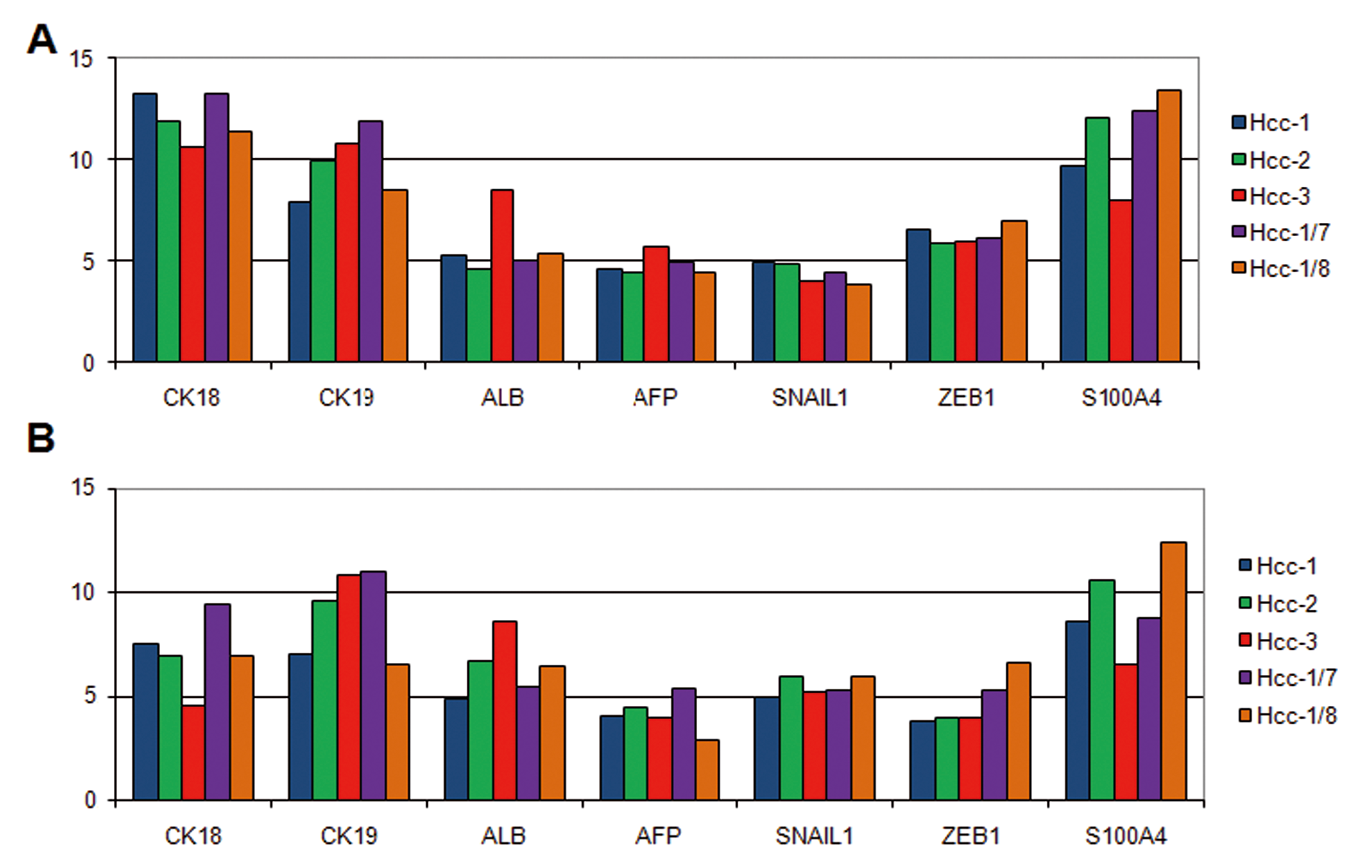

Supplement: Figure S4 — Gene expression validation. A) Microarray and B) qPCR expression values of selected genes in the cell lines and clones. The bars represent the mean relative expression values of three independent RNA isolations analysed in triplicate. For the qPCR analyses, all of the genes were normalised to CYC as the reference housekeeping gene. All of the data are presented as log2 transformations of gene-normalised signals. The qPCR results showed the same consistent modulation as the microarray analysis. (TIF) [file pone.0021369.s004.tif]

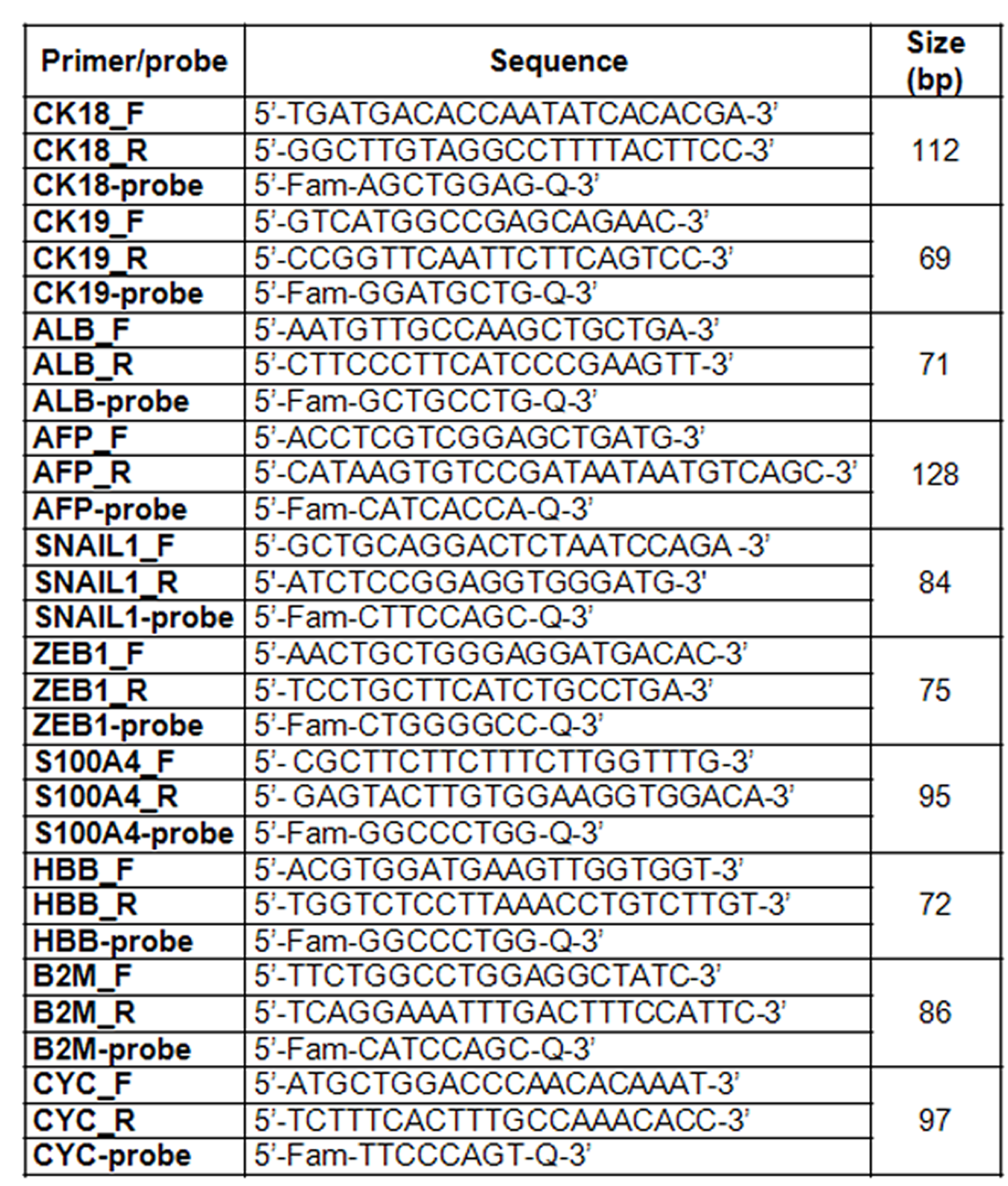

Supplement: Table S1 — Primer and probe sequences used in the qPCR experiments. (TIF) [file pone.0021369.s005.tif]
