# Supplementary material for: Evidence of Distinct Tumour-Propagating Cell Populations with Different Properties in Primary Human Hepatocellular Carcinoma
Source: PLoS One. 2011 Jun 23;6(6):e21369. doi: 10.1371/journal.pone.0021369 (PMC3121782; doi:10.1371/journal.pone.0021369)
Supplement: Text S1 — Supplementary Materials and Methods. (DOC) [file pone.0021369.s006.doc]

**Supplementary Materials and Methods**

**Fold increase**

**log 10**

*Gene expression analyses*

Total RNA was extracted starting from 0.5-1 x 10^6^ HCC cells and clones using the RNase Micro Kit (Qiagen, Crawley, UK). Five independent extractions were made for each cell population and, after checking the quality, concentration and integrity of the purified RNA, only the samples showing the best results were selected for further processing. The samples were hybridized on HumanWG-6 v3 Expression BeadChip (Illumina, San Diego, CA, USA) in accordance with the manufacturer’s instructions. After hybridization and washing, each array is scanned by Illumina scanning software (BeadScan) to produce a TIFF image. The probe level data were extracted from TIFF files using BeadScan, and then converted to normalized expression values using R and Bioconductor tools. Specifically, after the acquisition of the matrix of raw intensity data with the corresponding annotations, probes corresponding to “predicted” genes or not associated to a Entrez ID were discarded from the analysis; moreover, different probe signals corresponding to the same Entrez ID were converted into unique intensity signal, corresponding to the median of the values for each array, in order to obtain a single signal for each Entrez ID. After this process, we generated a boxplot of 16964 intensity signals whose values were log2-transformed and quantile normalized using the *aroma.light* package for Bioconductor [18]. Pearson correlation and average linkage were used as metrics for conventional agglomerative hierarchical clustering procedure in R software. The unsupervised analysis was performed on a subset of genes whose average change in expression levels varied at least 2-fold from the mean across the whole panel. DNA-Chip Analyzer (dChip) software was used for visualization [18]. Supervised analyses were performed using the RankProd package for Bioconductor, setting the value of pfp = .05 as threshold for significance. Venn diagram was built using the *ABarray* package for Bioconductor.

For qPCR, total RNA was isolated from the cells and clones using the High Pure RNA isolation Kit (Roche Applied Science). The cDNA underwent quantitative real-time PCR amplification with specific primers and probes for the keratin 18 (KRT18), keratin 19 (KRT19), albumin (ALB), alpha-fetoprotein (AFP), SNAIL1, zinc finger E-box binding homeobox 1 (ZEB1), S100A4, beta-2-microglobulin (B2M), cyclophilin A (CYC), and beta-globin (HBB) genes (Supplementary Table 1). The amplification was carried out on a LightCycler 2.0 System (Roche Applied Science). All of the reactions were performed in triplicate. All of the primers used had PCR efficiencies of >90%. The target genes were normalised to both B2M and CYC, and quantified using the comparative CT method (ΔΔCt) [19].
